# Study of the effect of surface oxidation treatment on the dielectric properties of delignified material

**DOI:** 10.1039/d5ra01052g

**Published:** 2025-04-22

**Authors:** Hui Xu, Fengqi Qiu, Weishuai Han, Jinwei Liu, Zhenhua Xue

**Affiliations:** a College of Material Science and Art Design, Inner Mongolia Agricultural University Hohhot 010018 China; b Key Laboratory of Fibrillation and Energy Development and Utilisation of Sandy Shrubs in Inner Mongolia Hohhot 010018 China; c National Forestry and Grassland Engineering Technology Research Centre for Efficient Development and Utilisation of Sandy Shrubs Hohhot 010018 China

## Abstract

To investigate the relationship between the degree of surface oxidation of delignification materials and their dielectric properties, sodium periodate was used to oxidize the delignification samples of *Pinus sylvestris* var. Mongolia and *Paulownia fortunei* (Seem.) Hemsl. The degree of oxidation was reflected by the degree of substitution of aldehyde and carboxyl groups at a specific reaction time interval, and then the dielectric constant and dielectric loss tangent were measured. The results show that with the increase of oxidation time, the crystallinity decreases and the hydrogen bond network is weakened. In the frequency range of 10^3^–10^7^ Hz, the dielectric constant of the sample decreases with the increase of frequency and tends to be stable near 10 MHz, but the dielectric constant of the oxide material is significantly higher than that of the untreated material. At the same time, the dielectric constant increases with the increase of oxidation time, while the dielectric loss tangent decreases.

## Abstract 2

To investigate the relationship between the degree of surface oxidation of delignification materials and their dielectric properties, sodium periodate was used to oxidize the delignification samples of *Pinus sylvestris* var.

## Graphical abstract

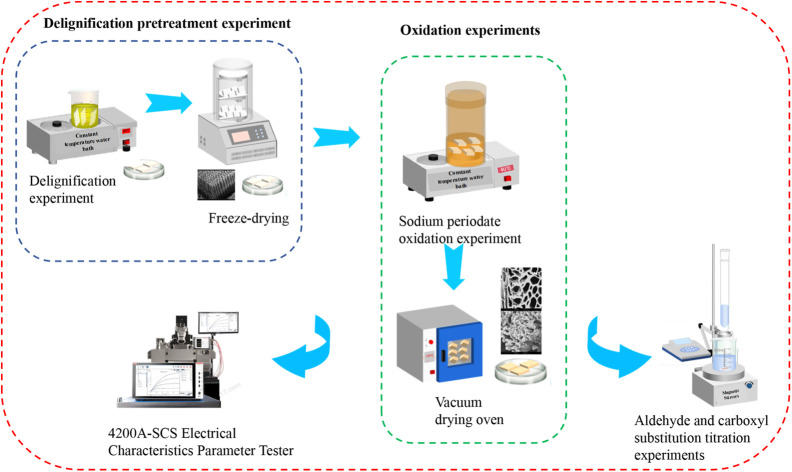

## 1. Introduction

Wood is a natural polymer composite material with a complex structure composed of different cells. It has a multi-scale hierarchical structure and can be used as an excellent modified basic material. Its internal cellulose is used as its own supporting basic material. Cellulose forms a rigid, linear cellulose chain at the molecular level through covalent bonds and hydrogen bonds during molecular entanglement^1,2^ Natural cellulose belongs to cellulose type I, with parallel chain orientation and a high proportion of hydroxyl groups inside. The van der Waals forces^3^ of intermolecular and intramolecular covalent bonds and strong hydrogen bonds of hydroxyl groups form a highly ordered crystal structure, and other unbonded parts form an amorphous region. The lone electron^4^ of the oxygen atom on the hydroxyl group gives cellulose a very high electron-donating effect. Therefore, the rich polar groups of cellulose and the excellent 3D framework structure of wood can be used as the basis for the optimization of dielectric function modification. Dielectric materials are important basic materials for capacitors. In common electronic devices, they generally have the function of storing energy. The dielectric properties of their materials directly affect their capacity and stability.

In the research field of dielectric materials, the existence of hydrogen bond network and glycosidic bond formed between and within the cellulose molecular chains makes the cellulose monomer form a chair conformation. The hydroxyl groups on each carbon atom are flat bonds, which makes the untreated cellulose weak in the electric field polarization response. However, the high proportion of hydroxyl groups gives it a large number of dipoles and a strong basis for electron-donating ability. Cellulose and its derivatives are one of the important raw materials for the study of cellulose functional materials. By introducing chemical and physical binding between cellulose molecules to change its spatial structure, other groups are used to replace polar groups and produce substituted cellulose derivatives, such as methylcellulose, hydroxypropyl cellulose (HPC), hydroxypropyl methylcellulose (HPMC), *etc.* Zhang^5^ and Lingbing Hu^6,7^ used the ' molecular channel engineering ' method to introduce Cu and Na ions into the molecular chains of coordination cellulose, destroy its hydrogen bond connection, release many hydroxyl groups, and increase the surface charge density of the sample. At the same time, the insertion of ions makes the hydroxyl group have more free volume, which promotes more dipole rotation and polarization in response to the external electric field. However, the oxidation of cellulose is usually studied by using an alkali-urea system to dissolve regenerated cellulose as a carrier, and regenerated cellulose is generally cellulose type II crystal, and its monoclinic crystal system is the most stable state in thermodynamics. However, due to the arrangement and accumulation of two adjacent cellulose chains in the unit cell in the form of anti-parallel,^8^ its electronegativity is weak. Due to the polarization of Maxwell Wagner Siller, dipoles interact with each other and hinder the polarization shift of polar groups in the electric field. Therefore, the oxidation treatment of cellulose can weaken the hydrogen bond network to a certain extent, increase the polarization phenomenon, improve the polarization mobility, and optimize the dielectric polarization phenomenon.

At present, although there are studies on the dielectric constant (*ε*′) of cellulose derivatives by regeneration oxidation and group substitution modification of cellulose, the effect of oxidation treatment of delignification wood (DW) on the dielectric properties of oxidized cellulose (OC) in different frequency ranges based on retaining the 3D framework tubular structure of wood has not been retrieved. Especially in the field of electronics and nanotechnology, wood can be used as a new type of green electronic product substrate material, and OC can be used as a modified carrier of electrical function reinforcement, which may contribute to its insulation or energy storage direction. Therefore, it is necessary to repeatedly analyze the coordination mechanism of the internal chemical composition of DW to OC materials and its dielectric properties.

Therefore, this study analyzed the mechanism of dielectric properties of oxidized cellulose after different oxidation times by using delignification materials for oxidation treatment. The *ε*′ and dielectric loss factor (tan*δ*) were measured at different frequencies (*F*), and the influence of oxidation degree on dielectric properties was analyzed, which provided effective data and a theoretical basis for the preparation of wood-based dielectric materials.

## 2. Experimental process

**Fig. 1:**
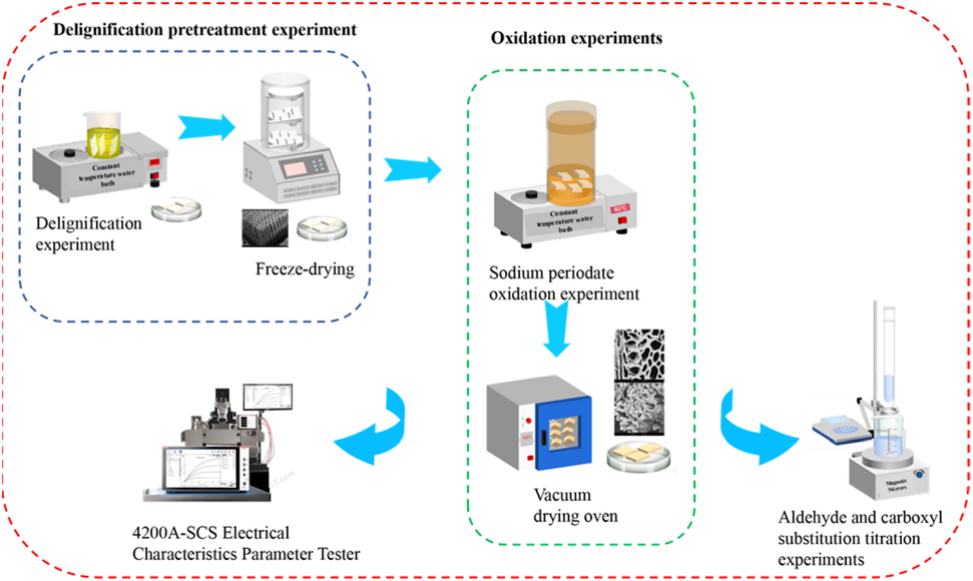
Test flow chart.

In this experiment, artificial *Pinus sylvestris* var.mongolica and *Paulownia fortunei* (Seem.) Hemsl. were used as raw materials. Firstly, delignification pretreatment was carried out by acid sodium chlorite method for 10 h, and then the pretreated wood was oxidized by sodium periodate method. Finally, the dielectric properties of the treated samples were tested. The specific test process is shown in Fig. 1.

### 2.1 Materials and reagents

Artificial camphor pine (*Pinus sylvestris* L. var. mongolica Litv.) and *Paulownia fortunei* (Seem.) Hemsl. were procured from a commercial source, subsequently processed into small specimens measuring 20 mm × 20 mm × 2 mm, and designated as pine (*Pinus sylvestris* L. var. mongolica Litv.) (M) and *Paulownia fortunei* (Seem.) Hemsl.) (P), respectively. The cellulose content of the camphor pine specimens post-delignification of the M was 76.75%, while that of the P was 77.25%.

Sodium periodate and 80% sodium chlorite were procured from Shanghai Aladdin Biochemical Technology Co., Ltd; glacial acetic acid, anhydrous ethanol, hydrochloric acid and sodium hydroxide were obtained from Tianjin Windship Chemical Reagent Technology Co. It should be noted that all the aforementioned chemicals were analytically pure, with distilled water being prepared *in situ* in the laboratory.

### 2.2 Instruments and equipment

Electrothermal Constant Temperature Triple Water Tank SHHW21-420, Shanghai Benting Instrument Co., Ltd; Universal High Speed Pulveriser (DE-150 g), Zhejiang Hongjingtian Industry & Trade Co., Ltd; Electrothermal Constant Temperature Blast Drying Oven GZX-GF101-1-BS-II/H, Shanghai Yuejin Medical Instrument Co., Ltd; Vacuum Drying Oven DZF-6020, Changzhou Jintan Jindaxi Instrument Manufacturing Co; Circulating Water Vacuum Pump SHZ-D(III), Shanghai Dongxi Refrigeration Instrument & Equipment Co., Ltd; Analytical Balance BS224S, Sartorius, Germany; Constant Temperature and Humidity Chamber JS-225L, Wuxi Tengchuan Instrument & Equipment Co., Ltd; pH Meter (FE28-Standard), Qingdao Lixi Bio-technology Co., Ltd; Ultra-thin Slicer EMUC7, Leica, Germany; 4200A-SCS Electrical Characteristics Parameter Tester, Keithley, Keithley Co.

### 2.3 Test methods

#### 2.3.1 Oxidation test

The delignification-treated wood samples were placed in a drying oven at 105 °C for 24 hours to ensure absolute drying treatment. After weighing, they were placed in a brown beaker, and 200 mL distilled water and a certain amount of NaIO_4_ were added (the mass ratio of the sample to sodium periodate was 1 : 2). The pH value of the reaction solution was adjusted to 2 with 0.1 mol L^−1^ HCl, and the reaction was carried out at 40 °C for a certain time under dark conditions.^9^ The reaction time variables were controlled at the set temperature and time, and the *Pinus sylvestris* Linn. var. mongolica Litv. and *Paulownia fortunei* (Seem.) Hemsl.s were controlled to design 6 groups of experimental time (1,2,3,4,5 and 6 h). After the treatment, 5 mL ethylene glycol^10^ was added to cool to room temperature for 2 h. After the reaction, it was washed with anhydrous ethanol 3 times and then washed with distilled water to neutralize it. Finally, it was dried at 50 °C for 24 h in a vacuum drying oven to obtain oxidized samples (OC) after different reaction times. The *Pinus sylvestris* var. Mongolia and *Paulownia fortunei* (Seem.) Hemsl. are denoted as M–OC–*x* and P–OC–*x* (*x* is the reaction time), respectively.

### 2.4 Testing and characterisation

#### 2.4.1 Determination of degree of substitution

##### 2.4.1.1 Determination of aldehyde content

According to ref. 11, the aldehyde content of OC samples after different reaction times was determined by the hydroxylamine hydrochloride method. The hydroxylamine hydrochloride aqueous solution reacted quantitatively with the aldehyde group to form oxime, and the released hydrochloric acid was titrated with standard sodium hydroxide aqueous solution. The reaction formula is as follows:–CHO + NH_2_OH·HCl → –CHNOH + HCL + H_2_OHCl + NaOH → NaCl + H_2_O

Add 0.1 g carboxymethyl chitosan (as a blank sample) and 30 mL distilled water into a conical flask, the volume of 100 mL, using pH meter to detect and record the corresponding data, 2 mL of 0.05 mol mL^−1^ hydroxylamine hydrochloride solution was reacted at room temperature for 4 h. Subsequently, 8 drops of thymol blue indicator were added, and the 0.05 mol L^−1^ sodium hydroxide solution was titrated into a conical flask until the solution showed blue and did not fade within 15–20 s. At the same time, the corresponding data detected and recorded by the pH meter were close to the results before the reaction. The same method was used to determine the volume of sodium hydroxide standard solution consumed by OC samples with different reaction times (1, 2, 3, 4, 5, and 6 h). Each group of experiments was repeated and three data calculations were read. The aldehyde content is calculated by the formula (1).1
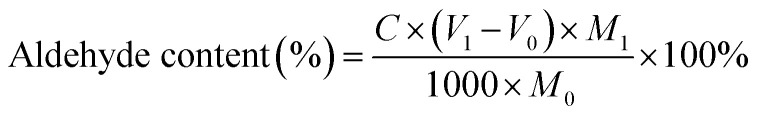


In the formula: the aldehyde group content is the number of aldehyde groups contained in 100 glucose units (%); *c* is the concentration of sodium hydroxide standard solution (mol L^−1^); *v*_1_ is the volume (mL) of sodium hydroxide standard solution consumed at different reaction times; *v*_2_ is the volume (mL) of blank carboxymethyl chitosan consuming sodium hydroxide standard solution; *m*_0_ is the mass of the absolute dry sample (g); *m*_1_ is the molar mass of a glucose unit, which is 161 g mol^−1^.

##### 2.4.1.2 Measurement of carboxyl group content

According to ref. 12–14, the carboxyl content of OC samples after different reaction times was determined by the sodium hydroxide-calcium acetate method. Firstly, the carboxylic acid was hydrochloric acidized by hydrochloric acid solution to eliminate the influence of metal ions in OC. Then, calcium acetate was used to exchange ions with the carboxyl group in OC. Finally, the carboxyl group was calculated by the amount of acetic acid released by the cation exchange of alkali titration. The reaction is as follows:–2COOH + (CH_3_COO)_2_Ca → (–COO)_2_Ca + 2CH_2_COOHCH_3_COOH + NaOH → CH_3_COONa + H_2_O

First, some samples were powdered, soaked in 0.1 mol mL^−1^ hydrochloric acid for 40 minutes, and then washed with distilled water to no chloride ion, filtered, and dried. Weigh 0.1g sample with 2wt% calcium acetate solution 10 mL impregnated in brown bottles, placed at room temperature for 15 h, and oscillation; phenolphthalein was used as a color indicator and titrated with 0.02 mol L^−1^ sodium hydroxide standard solution until the color of the solution changed to rose red and did not fade within 15–20 s. The same method was used to determine the volume of sodium hydroxide standard solution consumed by OC samples with different reaction times (1, 2, 3, 4, 5, and 6 h). Each group of experiments was repeated and the data were read three times, and the carboxyl content was calculated using formula (2).2
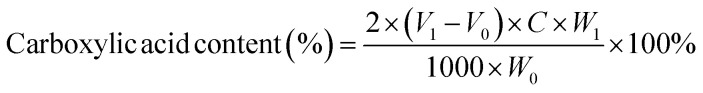


In the formula: carboxyl content is the molar number of carboxyl groups per unit mass, (mol g^−1^); *c* is the concentration of sodium hydroxide standard solution, (mol L^−1^); *v*_1_ is the volume (mL) of sodium hydroxide standard solution consumed by OC samples at different reaction times; *v*_2_ was the volume (mL) of sodium hydroxide standard solution consumed in the blank group; *w*_0_ is the mass of the dried sample (g), and *W*_1_ is the molar mass of a carboxyl unit, which is 45 g mol^−1^.

#### 2.4.2 Analytical tests

##### 2.4.2.1 Scanning electron microscopy (SEM)

The morphology of the OC samples was characterised at different reaction times. The samples were processed using a homogeneous ultrathin slicer (EMUC7), and the microscopic morphology changes were observed in the cross-section and radial section, respectively. The samples were sprayed with gold on the surface and then observed using a Phenom Pro SEM at an accelerating voltage of 15 kVv.

##### 2.4.2.2 XRD analysis

A D8advance X-ray diffractometer was utilised to ascertain and examine the crystalline alterations of OC samples at varying reaction times. These reaction times were established at ambient temperature, with a scanning range of 10–50 °C. The crystallinity of the samples was obtained by Jade5.0 software and the Segal method. Furthermore, the cellulose crystalline peaks were analysed by peak splitting and fitting using the Gaussian peaks method.

##### 2.4.2.3 FTIR characterisation

Alterations in the functional groups of the OC samples were analysed by means of the INVENIO S Fourier Transform Infrared Spectrometer for varying reaction times. The tests were carried out in potassium bromide compression mode.

#### 2.4.3 Determination of dielectric properties

The primary objective of the test was to ascertain the dielectric properties of the OC samples by employing the 4200A-SCS electrical characteristic parameter tester at a frequency range of 10^3^–10^7^ Hz. The test facilitated the determination of the curves of the dielectric constant (*ε*′) and the tangent of the dielectric loss (tan*δ*), in addition to the change of the conductivity (*σ*) of the samples with the frequency.

The capacitance value (*C*_p_) and loss angle tangent (tan*δ*) of the samples after different reaction times were determined, and their dielectric constants (*ε*′) and dielectric loss angle tangent (tan*δ*) were converted by using the equations given in (3) and (4). It was established that there is a certain correlation between the conductivity of the samples and the relative dielectric loss^15^ of the medium. The conductivity (*σ*) of the samples was obtained by repeating the readings three times and taking the average value.3
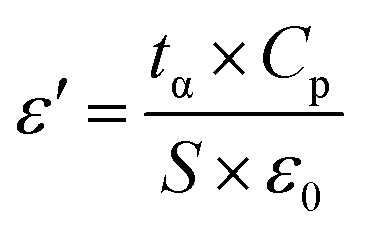
where: *t*_α_ average thickness of the material (m); *C*_p_ capacitance value (*F*); *S* electrode area (m^2^); *ε*_0_ vacuum capacitance (8.854 × 10^−12^ F m^−1^).4
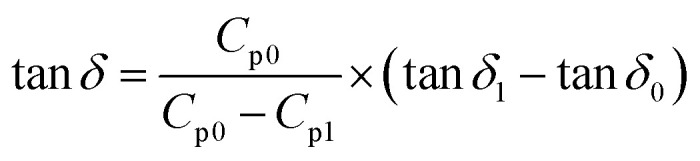
where: capacitance value (*F*) before *C*_p0_ is put into OC; capacitance value (*F*) after *C*_p1_ is put into OC; tangent of loss angle after tan*δ*_1_ is put into OC; tangent of loss angle before tan*δ*_0_ is put into OC.

5 *σ* = *ε*_0_ × *ε*′ × *ω* × tan*δ*

where: *σ* is the conductivity (S m^−1^); *ω* is the angular frequency (rad s^−1^); *ω* = 2πf, *f* is the frequency (Hz); *ε*_0_ vacuum capacitance (8.854 × 10^−12^ F m^−1^); *ε*′ is the dielectric constant of the medium.

## 3. Results and analyses

### 3.1 Effect of reaction time on OC morphology analysis

**Fig. 2:**
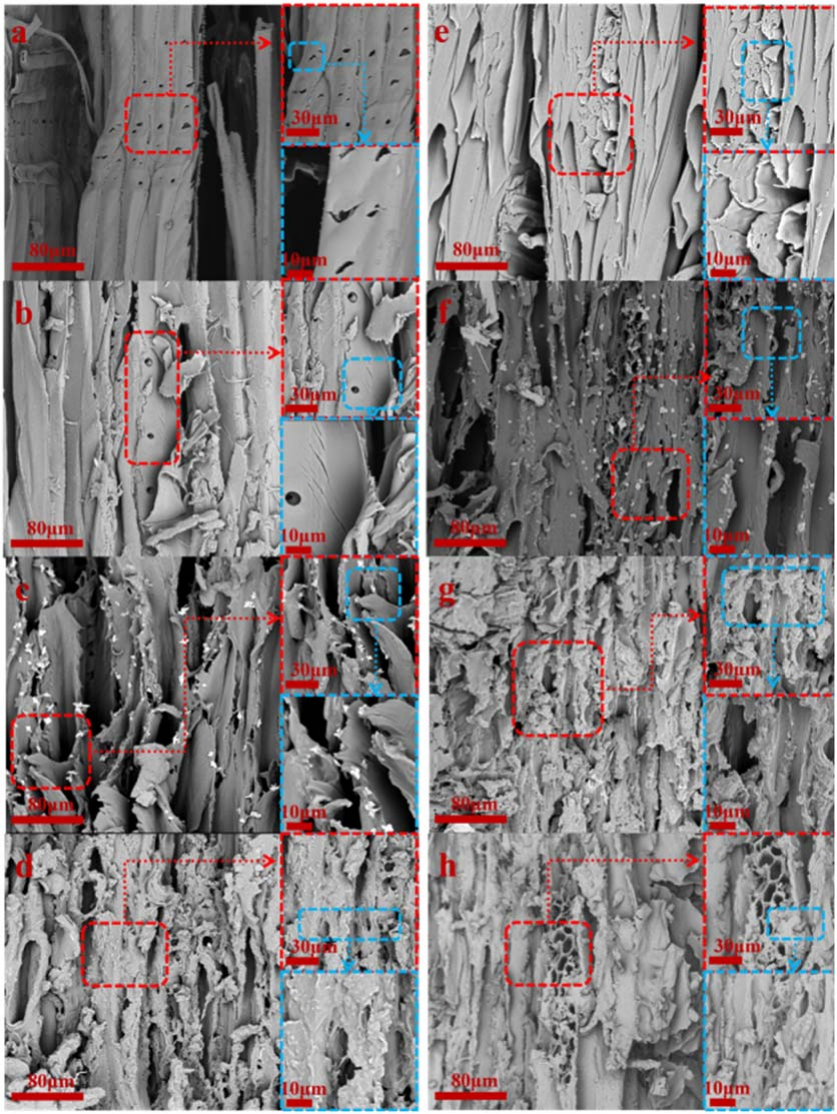
Changes in diameter cross-section before and after oxidation experiment of *Pinus sylvestris* var. mongolica and paulownia wood (a) M–OC–0; (b) M–OC–1; (c) M–OC–3; (d) M–OC–6; (e) P–OC–0; (f) P–OC–1; (g) P–OC–3; (h) P–OC–6.

**Fig. 3:**
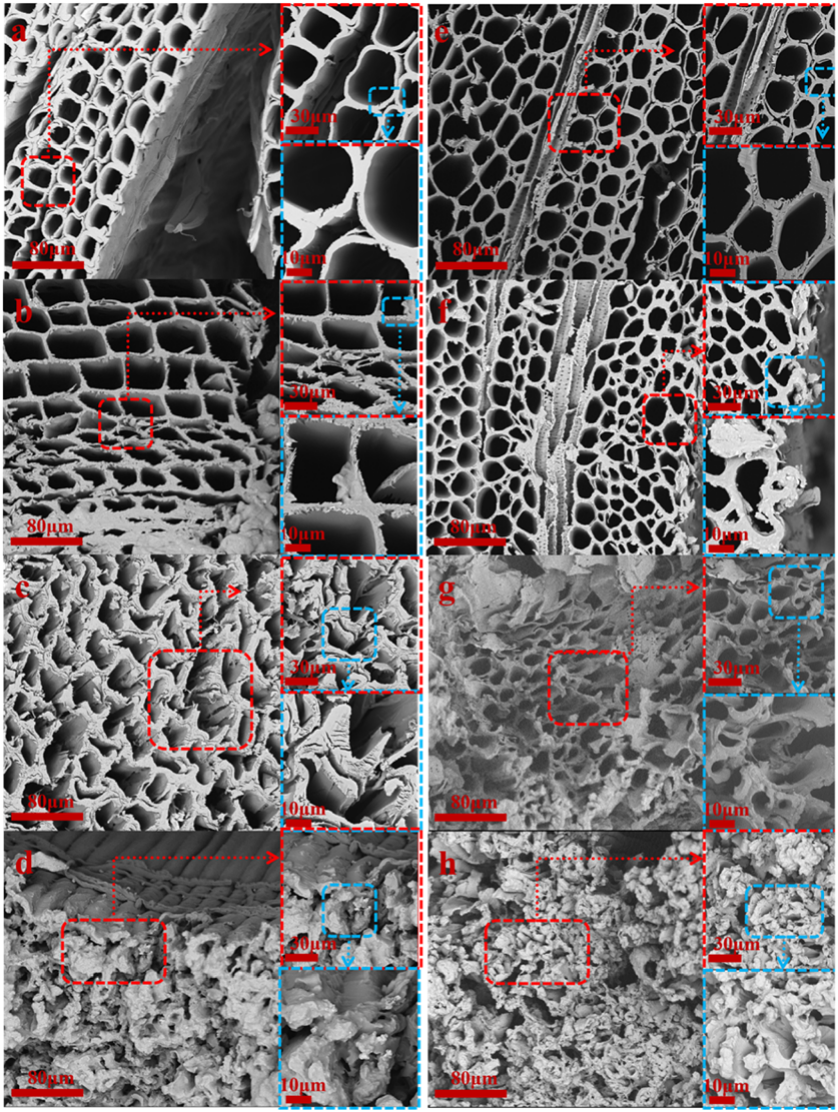
Cross sectional changes of *Pinus sylvestris* var. mongolica and paulownia before and after oxidation experiment. (a) M–OC–0; (b) M–OC–1; (c) M–OC–3; (d) M–OC–6; (e) P–OC–0; (f) P–OC–1; (g) P –OC–3; (h) P–OC–6.

In order to confirm the effect of oxidation time on the surface structure and hollow cavity size of delignification wood, the results of the SEM test are shown in Fig. 2 and 3.

It can be seen from Fig. 3 that the surface of the sample collapsed after oxidation with sodium periodate. It can be inferred that the hydrogen bond network connected by the hydroxyl groups between the cellulose molecules was broken, and its relatively neatly arranged order degree had a certain degree of relief.^16^ With the increase in reaction time, the burr phenomenon of the sample was obvious, and the surface roughness increased.

Although the types of wood are different, the results are shown in Fig. 2: for the cross-section, with the increase of oxidation time, the cell wall of the cross-section of the tube hole section is loose and expanded, but there are obvious cracks in the surrounding cell wall, the tube hole of the cross-section is collapsed, and the diameter of the tube hole is reduced. In Fig. 3, for the radial section, with the increase of oxidation time, the surface roughness of the sample increased significantly, and there were some cracks in the pore wall of the tube. The cellulose on the surface was cracked to a certain extent, and some burrs were produced. The cracks increased significantly. At the same time, the reaction time was close to 3 h, and the section was found to have obvious cracking. The fracture began to change from toughness to brittleness. At the reaction time of 6 h, it was found that the cell wall damage was more obvious, but it could maintain the basic 3D framework. It was found that the cellulose oxidation part accumulated on the surface of the cell wall at the cross-section and the radial section.

### 3.2 Effect of reaction time on the degree of OC oxidation

**Fig. 4:**
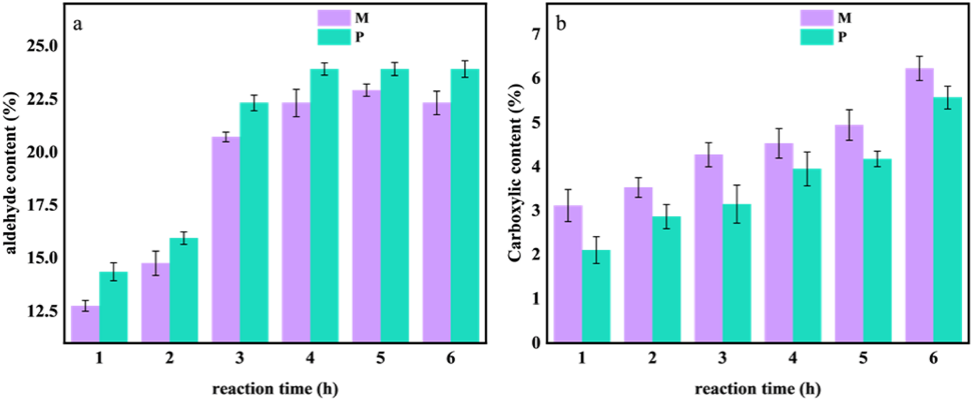
The changes in aldehyde and carboxyl content during the 0–6 hours oxidation experiment of *Pinus sylvestris* var. mongolica and paulownia wood. (a) Aldehyde content; (b) carboxyl content.

In order to explore the degree of oxidation of cellulose in OC samples at different reaction times, the degree of substitution of the aldehyde group and carboxyl group was calculated according to the method in 1.4.1. The results are shown in Fig. 4.

It can be seen from Fig. 4a that with the increase in reaction time, the degree of oxidation of the sample gradually increased. Compared with different tree species, when the reaction time increased from 0 h to 6 h, the aldehyde content of M increased from 12.75% to 22.31%, and the aldehyde content of P increased from 14.35% to 23.91%. When the treatment time reached 4–6 h, the aldehyde content of the sample increased steadily, and the aldehyde content of P was higher than that of M; in Fig. 4b, when the reaction time increased from 0 h to 6 h, the carboxyl content of M and P increased with the increase of reaction time. The carboxyl content of M increased from 3.12 mol L^−1^ to 6.22 mol L^−1^, and the carboxyl content of P increased from 2.10 mol L^−1^ to 5.56 mol L^−1^.

It can be seen that during the oxidation process of the sample, the hydroxyl groups on the cellulose molecular chain in the OC sample are oxidized by sodium periodate as an oxidant, and some of their hydroxyl groups are oxidized to aldehyde groups and carboxyl groups so that the hydrogen bond network constructed within and between the molecules of the cellulose is oxidized. The hydroxyl group weakens its van der Waals force.

### 3.3 Effect of reaction time on OC crystallinity

**Fig. 5:**
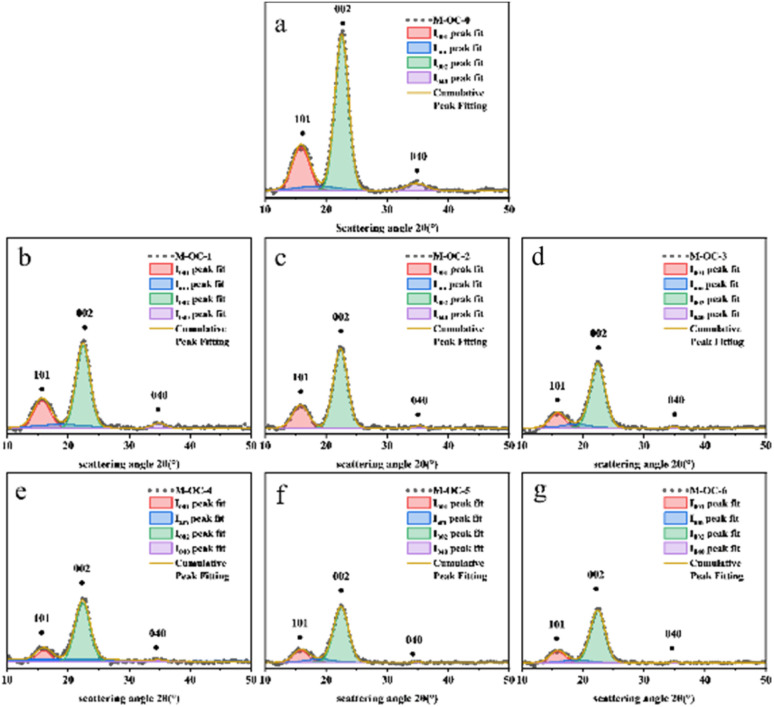
X-ray diffraction patterns of *Pinus sylvestris* var. mongolica before and after oxidation experiment (a) M–OC–0; (b) M–OC–1; (c) M–OC–2; (d) M–OC–3; (e) M–OC–4; (f) M–OC–5; (g) M–OC–6.

**Fig. 6:**
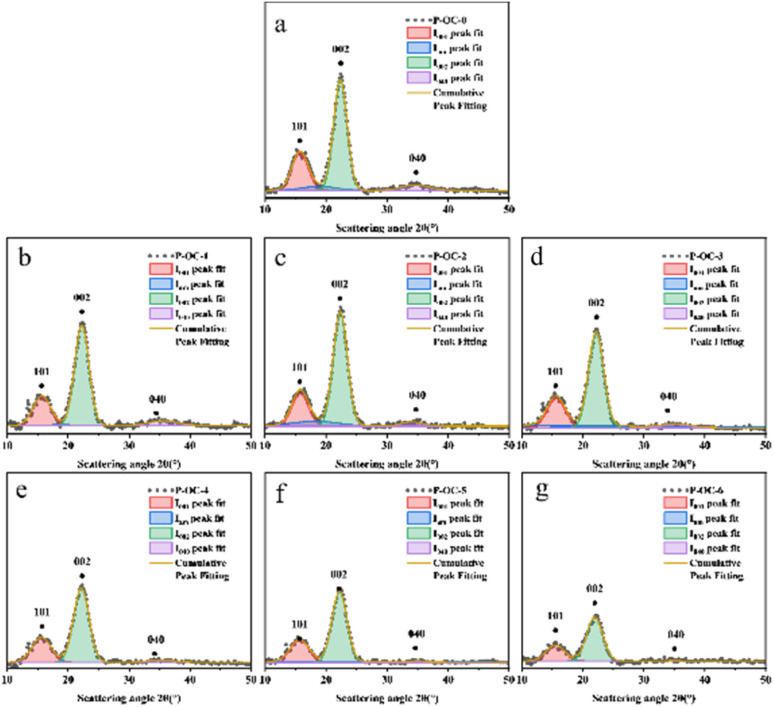
X-ray diffraction patterns of paulownia wood before and after oxidation experiment (a) P–OC–0; (b) P–OC–1; (c) P–OC–2; (d) P–OC–3; (e) P–OC–4; (f) P–OC–5; (g) P–OC–6.

**Fig. 7:**
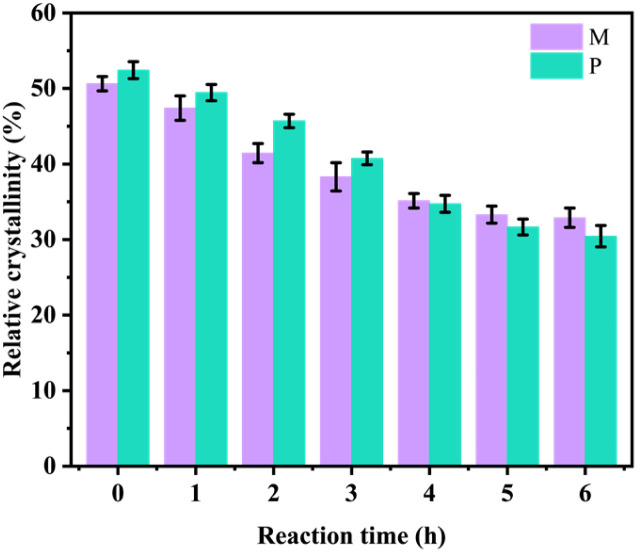
Crystallinity changes of *Pinus sylvestris* var. mongolica and paulownia during 0–6 hours of oxidation experiment.

With the increase of oxidation time, the content of the aldehyde group and carboxyl group in M and P increased. The strong hydrogen bond interaction of the hydroxyl group and van der Waals force made the cellulose molecules form a highly ordered crystal structure.^17^ However, with the increase of oxidation degree, the hydrogen bond network of the sample was weakened and its crystal structure was destroyed. To verify this result, an X-ray diffractometer was used to analyze the changes in X-ray diffraction patterns before and after M and P oxidation treatment. The results are shown in Fig. 5–7.

From Fig. 5 and 6, it can be seen that the crystal structure peak shape of cellulose did not change after oxidation treatment at different reaction times. Gaussian peaks method^18–20^ peak fitting treatment can be seen that all samples have typical X-ray diffraction characteristics of cellulose type I, and its (101), (002), and (0 40) typical characteristic crystal planes. It was found that the characteristic crystal plane peak of cellulose in OC samples decreased significantly with the increase of reaction time, especially the changing trend of crystal peak–peak value in (040) was more obvious.

By the Segal method, it was found that when the reaction time increased from 0 h to 6 h in Fig. 7, the crystallinity of M decreased from 50.63% to 32.88%, and the crystallinity of P decreased from 52.43% to 30.45%. The above analysis is consistent with the conclusion of 2.2, which can prove that the hydroxyl group of cellulose is oxidized to the aldehyde group and carboxyl group, and its oxidation reaction will weaken the hydrogen bond network composed of the hydroxyl group and reduce the crystallinity of OC sample.

### 3.4 Effect of reaction time on the molecular structure of OC samples

**Fig. 8:**
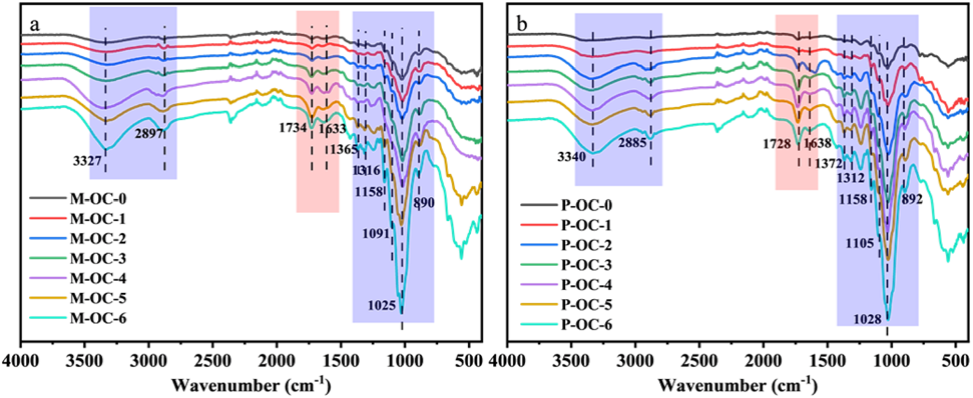
Infrared spectra of *Pinus sylvestris* var. mongolica and paulownia before and after oxidation experiment (a) M; (b) P.

According to 2.2 and 2.3, it can be determined that with the increase of oxidation time, the hydroxyl groups in M and P are oxidized and the hydrogen bond network is weakened. Therefore, FT-IR is used to analyze the changes of chemical functional groups before and after oxidation treatment of M and P, as shown in Fig. 8. The corresponding attribution relationship of infrared absorption signals is shown in Table 1.

**Table 1:** Attribution of infrared absorption signals of *Cinnamomum camphora* and *Pterocarpus indicus*

M absorption signal cm^−1^	P absorption signal cm^−1^	Functional groups
3327	3340	Mainly –OH stretching vibrations in the free and bound water fractions
2891	2885	–CH_3_, –CH_2_ stretching vibrations of cellulose
1734	1728	The –C=O stretching vibration peak of a non-conjugated acetyl or acid group
1633	1638	–C=O induced telescopic vibrations
1365, 1316	1372, 1312	C–H absorption peaks of aliphatic methyl and ether hydroxyl groups
1158, 1097	1158, 1105	Stretching vibrations of the –C–O bond of cellulose
1025	1028	Vibrations induced by –C–O–C and –C=O of cellulose and hemicellulose
890	892	β-Glycosidic bond vibrations of cellulose stretching vibrations of glycosidic bonds

As demonstrated in Fig. 8, an enhancement in the intensity of specific characteristic peaks in M and P is observed with an increase in oxidation time. Fig. 8a illustrates the peaks at 3327 cm^−1^ and 890 cm^−1^ in M, which correspond to the expansion and contraction vibrations of the –OH^21^ groups in both the free and bound water portions of the OC samples and the expansion and contraction vibration of the vibrational glycosidic bond of β-glycosidic bond^22^ of cellulose, respectively, but the position of them stays unchanged, and the same elevation trend is also observed for P. It can be seen that the hydroxyl peaks have an obvious tendency to elevate and broaden, which proves that the number of hydroxyl groups in the free portion increases during the oxidation process, which may be due to the weakening of the hydrogen bonding network, and the relative content of hydroxyl groups on the exposed cellulose surface increases, and the proportion of the amorphous region of the cellulose increases, which is in agreement with the analytical result of 2.3 that the crystallinity of the samples decreases with the increase of the oxidation time.

A subsequent comparison of the IR spectra before and after oxidation reveals the emergence of characteristic peaks at 1734 cm^−1^, 1633 cm^−1^ and 1091 cm^−1^, which are attributed to the oxid The –C=O stretching vibration peaks of the non-conjugated acetyl or acid groups, the stretching vibration induced by –C=O, and the stretching vibration of the –C–O bond of cellulose, correspond to the characteristic peaks^23–25^ at 1734 cm^−1^, 1633 cm^−1^ and 1091 cm^−1^, respectively, in oxidised cellulose samples such as M. Notably, the 1734 cm^−1^ peak corresponds to the C=O stretching vibration of the aldehyde group,^26^ which is a by-product of the oxidation of the o-diol at positions 2,3 on the cellulose main chain to a dialdehyde group by sodium periodate. This process also results in the formation of a hemiacetal from the aldehyde group and the adjacent hydroxyl group^27^ within the oxidised cellulose molecule. This outcome aligns with the findings of infrared spectroscopic analyses of dialdehyde starch^28^ and dialdehyde cellulose.^29^ As the oxidation reaction progressed, the out-of-plane vibration of O–H became apparent, suggesting the potential for further oxidation of the aldehyde group to a carboxyl group. This finding aligns with the observations presented in Figure 2.2, which showcases the variation in the content of aldehyde and carboxyl groups over time during the oxidation process. Additionally, Fig. 8b provides a comparative analysis of the changes in the content of these groups for P, further substantiating the conclusions drawn from the present study.

### 3.5 Effect of reaction time on the dielectric properties of OC samples

**Fig. 9:**
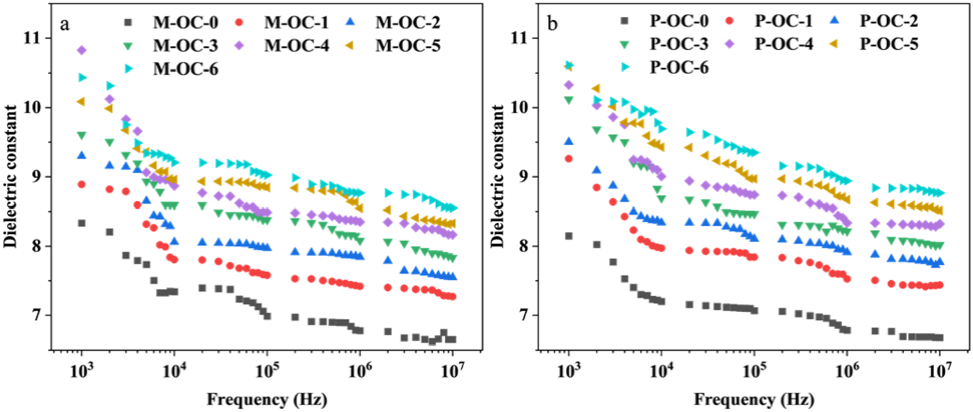
The variation of *ε*′ in OC samples of *Pinus sylvestris* var. mongolica and paulownia at different *F* values (a) M-*ε*′; (b) P-*ε*′.

**Fig. 10:**
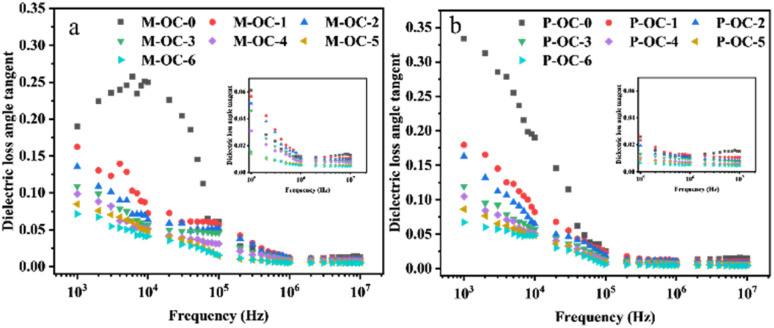
Changes in tan*δ* of OC samples of *Pinus sylvestris* var. mongolica and paulownia at different *F* levels (a) M-tan*δ*; (b) P-tan*δ*.

**Fig. 11:**
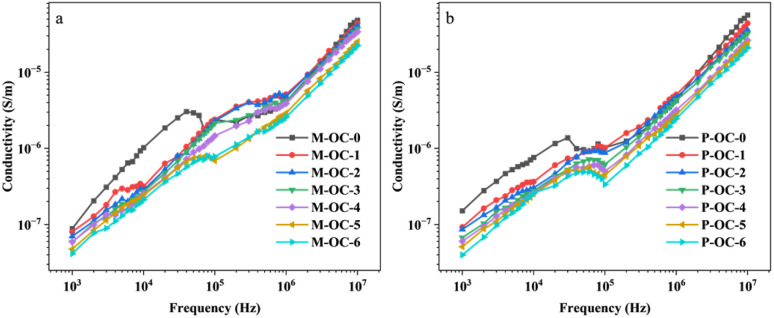
The variation of *σ* in OC samples of *Pinus sylvestris* var. mongolica and paulownia at different *F* values (a) M-*σ*; (b) P-*σ*.

The molecular-level assembly of cellulose in wood occurs through a non-centrosymmetric structure, characterised by covalent bonding, van der Waals forces and hydrophobic interactions between intra- and inter-chain hydrogen bonds. The arrangement of the reduced and non-reduced ends of all the cellulose chains is pivotal in this process. The hydroxyl groups in this non-centrosymmetric arrangement are capable of producing a net dipole moment,^30^ and the lone electron pairs of oxygen atoms on the hydroxyl group give it a higher electron-donating effect,^31^ meaning that it can be dipolarised under an electric field. Dipole polarisation can occur under the action of an electric field; however, the strong hydrogen bonding of the hydroxyl group in its crystal structure limits the polarisation shift in electric field. Consequently, the hydroxyl group of the sample was oxidised. In combination with 2.2–2.4, it is evident that an increase in oxidation time results in the degradation of the hydrogen bonding network within the sample, a reduction in the crystallinity of the sample, and the exposure of active groups, such as hydroxyl and carbonyl groups, present in cellulose. Concurrently, the hydroxyl group undergoes oxidation, whereby it is replaced by aldehyde and carboxyl groups. The presence of different substituent functional groups leads to varied electron-donating effects, which in turn influence the orientation shift in the AC electric field of OC samples. This phenomenon is further compounded by the oxidation process, which exerts a distinct effect on the dielectric properties of OC samples. The dielectric constant (*ε*′), dielectric loss angle tangent (tan*δ*) and conductivity (*σ*) of M−OC and P–OC at varying frequencies (*F*) and adiabatic conditions for different oxidation times were measured and calculated by using the 4200A-SCS Electrical Characteristic Parameter Tester, as shown in Fig. 9–11.

As demonstrated in Fig. 9, the frequency dependence of *ε*′ (*ω*) at varying oxidation times is exhibited. Fig. 9a and b reveal that *ε*′ increases with the prolongation of oxidation time within the frequency range of 10^3^–10^7^ Hz. It may be hypothesized that the highly ordered crystal structure composed of cellulose molecules is damaged during the oxidation reaction, resulting in weakened van der Waals forces and covalent bonds between its cellulose molecules. This leads to an increase in the proportion of active groups (primary hydroxyl, methylene and carbonyl groups)^32^ on the surface of its cellulose, an increase in the number of polaritons, and the oxidative substitution of hydroxyl groups leads to an increase in the dipole moment of the molecules, and the groups are more likely to orientate to form a new polar region in the presence of an applied electric field.^5^ The degree of freedom of the polar groups in the electric field polarisation response increases, which improves the dielectric responsiveness, so *ε*′ also rises.

Meanwhile *ε*′ decreases with the increase of *F*. As the oxidation time increases and the degree of oxidation of the samples increases, the trend of *ε*′ decreases more and more obviously, while *ε*′ of the oxidised samples is higher than that of the pre-treated material. This may be due to the fact that under an alternating electric field, it is found that at low frequencies, *ε*′ has a higher value inside the sample in due to the contribution of space charge^33^ and electronic polarisation.^34^ Then it decreases with increasing frequency, when the sample is mainly orientationally polarised internally. The decrease in *ε*′ may be caused by the molecular hysteresis behind the change in the applied electric field due to the dipole not being able to reach the maximum polarisation value under the corresponding electric field fast enough, resulting in a lower polarisation and therefore a decrease in *ε*′.

As demonstrated in Fig. 11, the calculation of the slope [d(log *σ*)/d(log *ω*)] from the frequency dependence of *σ*(*ω*) indicates a decrease in *K* with increasing oxidation time. This phenomenon can be attributed to the electric field-induced dissociation of polar groups within the sample.^35^ The p*K*_a_ value is typically in the range of 4–5 when the carboxyl group is attached to the alkyl group, and the p*K*_a_ values of the predominant phenolic structures^36^ with conjugated, non-conjugated carbonyl, and condensed structures are 7.7, 9.9, and 12.0 units, respectively, and those of alkane structures with sec-hydroxyl and primary hydroxyl groups are 15.5 and 15.9 units, respectively. It has been demonstrated that, in electric field polarisation, the primary source of polariton is subject to alteration, *i.e.* the dipole of the pre-polarisation phenomenon is derived from the surface active groups (sec-hydroxyl and primary hydroxyl) in the amorphous zone of cellulose. With the increase of oxidation time, the crystalline zone of the samples is damaged, the proportion of the amorphous zone rises, and at the same time hydroxyl is replaced by aldehyde and carboxyl, and the p*K*_a_ value of group^37^ is lower than the corresponding value of the hydroxyl group. The electronegativity of group is higher than that of hydroxyl. The increase in the mobility of polar groups increases the orientation polarisation, and the polarisation response in the electric field is stronger. Therefore, with the increase of oxidation time, the *ε*′ value of the OC samples is greater.

It has been established that the dielectric relaxation behaviour of polar groups in cellulose molecules is closely related to their motor activity and orientation kinetics under applied electric field. The frequency dependence of tan*δ* (*ω*) according to different oxidation times is shown in Fig. 10. As demonstrated in Fig. 10a and b, tan*δ* exhibits a decline in the frequency range of 10^3^–10^5^ Hz, accompanied by an increase in the frequency range of 10^5^–10^7^ Hz. Conversely, the value of tan*δ* exhibits a tendency to flatten out gradually with the increase of *F*. The frequency dependence of tan*δ* (*ω*) in the low frequency region is depicted in Fig. 10a and b, which is analogous to that in the low frequency region. It can be hypothesised that, within the low-frequency region, cellulose chain movement and space charge polarisation in the sample are increased, resulting in elevated tan*δ*. With an increase in the electric field frequency, the polarisation exhibits a time scale in response to the alternating electric field. Internal friction and mutual interaction during the movement of the dipoles.^38^ The polar group orientation polarisation mainly occurs in the sample, and the movement of its orientation tends to be stabilised in the high-frequency range, so that the tan*δ* shows a trend of decreasing and then stabilising.

As demonstrated in Fig. 10a and b, a comparison of tan*δ* changes at varying oxidation times is provided. As demonstrated in the figure, tan*δ* decreases with increasing oxidation time, and at higher frequencies, in the range of 10^5^–10^7^ Hz, the dipole is unable to follow the fast response of the electric field. With increasing oxidation, the aldehyde and carboxyl content of the samples increases, and the disruption of the hydrogen bonding network, and the dissolution of some of the cellulose in the OC samples occurs, as can be seen from the analysis in 2.1. The introduction of carboxyl groups with high dipole moments generally results in strong spatial site resistance^39^ and an overall limitation of the polarisation shift of the polariton orientation polarisation in the electric field. Substitution of hydroxyl groups on the surface of cellulose during oxidation may lead to (1) disruption of the pyranose ring on cellulose and partial substitution of the side groups, causing a change in the dipole moment of the cellulose molecular chain. (2), substitution of the oxidised cellulose side groups by more bulky functional groups has been observed, which increases the volume of the crosslinked structure formed with the remaining hydrogen bonds. However, the density of polaritons in the crosslinked space decreases, so that the tan*δ* value decreases with increasing oxidation time.

## 4. Conclusions

The oxidation degree of cellulose in wood is the main factor affecting its dielectric properties. The effects of different oxidation time on *ε*′ and tan*δ* of OC samples were studied by oxidizing the wood base of *Pinus sylvestris* var. mongolica and pine (*Pinus sylvestris* L. var. mongolica Litv.) after delignification treatment.

(1) With the increase of oxidation time, the crystallinity of the samples decreased. In addition, the hydrogen bond network was significantly weakened, while the content of aldehyde and carboxyl groups increased. The results showed that the aldehyde content was 23.12%, and the carboxyl content was 5.89%. With the extension of oxidation time, the mobility of aldehyde and carboxyl substituted hydroxyl groups and polar groups increased, and *ε*′ increased; a large number of positive groups hindered their freedom in the electric field, and tan*δ* showed a downward trend.

(2) In the discussion of dielectric properties, it is necessary to further explore the relationship between electrical parameters and the degree of oxidation of cellulose in the sample. Although it has been proposed that, contrary to hydroxyl, aldehyde and carboxylic acid groups are the main contributors to reduce the mobility of polar groups through the p*K*_a_ value of substituent groups, further research is needed to determine the extent to which different groups can replace the polar effects that affect the degree of freedom of movement. Further research is needed to explore the change rule of oxidized cellulose as matrix material, modified in the future to improve the dielectric parameters and the dielectric properties of the material.

## Author contributions

Xu Hui: writing – manuscript preparation; qiu Fengqi, writing comments and editing; han Weishuai: writing comments, data collation; liu Jinwei: writing inspection and editing; xue zhenghua: funding, writing-reviewing and editing, validation.

## Conflicts of interest

The authors declare no conflict of interest.

## References

[cit1] Lucenius J., José Valle-Delgado J., Parikka K., Österberg M. (2019). J. Colloid Interface Sci..

[cit2] Meng Y., Xia Y., Young T. M., Cai Z., Wang S. (2015). RSC Adv..

[cit3] Ram F., Garemark J., Li Y., Berglund L. (2022). Composites, Part A.

[cit4] Moon R. J., Martini A., Nairn J., Simonsen J., Youngblood J. (2011). Chem. Soc. Rev..

[cit5] Zhang P., Huang H., Wang X., Cai K., Chen J., Xu Y., Yu F., Nie S., Wang S., Liu X. (2024). Nano Energy.

[cit6] Yang C., Wu Q., Xie W., Zhang X., Brozena A., Zheng J., Garaga M. N., Ko B. H., Mao Y., He S., Gao Y., Wang P., Tyagi M., Jiao F., Briber R., Albertus P., Wang C., Greenbaum S., Hu Y.-Y., Isogai A., Winter M., Xu K., Qi Y., Hu L. (2021). Nature.

[cit7] Li T., Zhang X., Lacey S. D., Mi R., Zhao X., Jiang F., Song J., Liu Z., Chen G., Dai J., Yao Y., Das S., Yang R., Briber R. M., Hu L. (2019). Nat. Mater..

[cit8] Luo Q., Shen H., Zhou G., Xu X. (2023). Carbohydr. Polym..

[cit9] Kumar Mudedla S., Vuorte M., Veijola E., Marjamaa K., Koivula A., Linder M. B., Arola S., Sammalkorpi M. (2021). Cellulose.

[cit10] Xiao D., Jiang M., Luo X., Liu S., Li J., Chen Z., Li S. (2021). ACS Sustainable Chem. Eng..

[cit11] Ju T., Liu L., Jiang Y., Li H., Wei M., Chen Y., Mu G., Min D., Chen Z., Xie Y., Li J., Xiao S. (2024). ACS Sustainable Chem. Eng..

[cit12] Guo D., Ren W., Yao S., Li J., Yu Y., Chu F. (2024). ACS Nano.

[cit13] Zhou Y., Li X., Lv Y., Shi Y., Zeng Y., Li D., Mu C. (2016). Carbohydr. Polym..

[cit14] Liu P., Pang B., Dechert S., Zhang X. C., Andreas L. B., Fischer S., Meyer F., Zhang K. (2020). Angew. Chem., Int. Ed..

[cit15] Khviyuzov S., Bogolitsyn K., Volkov A., Koposov G., Gusakova M. (2020). Holzforschung.

[cit16] Jingxu H., Minwen L. I., Zhihan L. I., Haibo H. (2024). Acta Mater. Compos. Sin..

[cit17] Jiang K., Yan Z., Fang W., Zhang Y. (2022). Mater. Res. Express.

[cit18] Park S., Baker J. O., Himmel M. E., Parilla P. A., Johnson D. K. (2010). Biotechnol. Biofuels.

[cit19] Ju X., Bowden M., Brown E. E., Zhang X. (2015). Carbohydr. Polym..

[cit20] Ahvenainen P., Kontro I., Svedström K. (2016). Cellulose.

[cit21] Tang Z., Li W., Lin X., Xiao H., Miao Q., Huang L., Chen L., Wu H. (2017). Polymers.

[cit22] Wang K., Dong Y., Ling Z., Liu X., Shi S. Q., Li J. (2021). Compos. Sci. Technol..

[cit23] Kolya H., Kang C.-W. (2022). Carbohydr. Polym..

[cit24] Hwang J. H., Martinez D. V., Martinez E. J., Metavarayuth G., Goodlett D., Wang Qi, Ganewatta M., Kent M. S., Tang C. (2022). Giant.

[cit25] Yang C., Magallanes G., Maldonado S., Stephenson C. R. J. (2021). J. Org. Chem..

[cit26] Zhao F., Shi Ye, Pan L., Yu G. (2017). Acc. Chem. Res..

[cit27] Huntington B., Pitcher M. L., Sheikhi A. (2023). Biomacromolecules.

[cit28] Zhang W., Liu H., Chen Z., Yang Z., Zhang X., Wang X. (2024). ACS Appl. Mater. Interfaces.

[cit29] Park S., Park B.-D. (2022). J. Ind. Eng. Chem..

[cit30] Zhang Y., Zhang Z., Peng W., He J., Xu B., Song X., Li J., Luo Y., Nie S., Wang S., Liang D., Duan Q. (2024). Chem. Eng. J..

[cit31] Du G., Wang J., Liu Y., Yuan J., Liu T., Cai C., Luo B., Zhu S., Wei Z., Wang S., Nie S. (2023). Adv. Sci..

[cit32] Chen Y., Zhang L., Yang Y., Pang B., Xu W., Duan G., Jiang S., Zhang K. (2021). Adv. Mater..

[cit33] Shan X., Wu J., Zhang X., Wang Li, Yang J., Chen Z., Yu J., Wang X. (2021). Cell Rep. Phys. Sci..

[cit34] Wen H., Cheng L., Jiang Yi, Zhu T., Chen Z., Dai X., Gao C. (2021). J. Electr. Eng. Technol..

[cit35] Abdel-karim A. M., Salama A. H., Hassan M. L. (2018). J. Phys. Org. Chem..

[cit36] Bogolitsyn K. G., Khviuzov S. S., Gusakova M. A., Pustynnaya M. A., Krasikova A. A. (2018). Wood Sci. Technol..

[cit37] Liu Di, Zhou L., Cui S., Gao Y., Li S., Zhao Z., Yi Z., Zou H., Fan Y., Wang J., Wang Z. L. (2022). Nat. Commun..

[cit38] Li D.-L., Shi S.-C., Lan K.-Y., Liu C.-Y., Li Y., Xu L., Lei J., Zhong G.-J., Huang H.-D., Li Z.-M. (2023). ACS Macro Lett..

[cit39] Wang Y., Zhang H., Lin L., Wu R., Wang X., Ren J., Liu C., Wang X. (2020). Pap. Biomater..

